# Abnormally Increased Prolactin Levels in Women with Polycystic Ovarian Syndrome Are Associated with Risk of Obesity, Insulin Resistance and Prediabetes

**DOI:** 10.3390/ijms26094239

**Published:** 2025-04-29

**Authors:** Vesselina Yanachkova, Teodora Stankova

**Affiliations:** 1 Department of Endocrinology, Specialized Hospital for Active Treatment of Obstetrics and Gynecology “Dr. Shterev”, 1330 Sofia, Bulgaria; v_ess@abv.bg; 2 Research Institute, Medical University-Pleven, 5800 Pleven, Bulgaria; 3 Department of Medical Biochemistry, Faculty of Pharmacy, Medical University of Plovdiv, 4002 Plovdiv, Bulgaria

**Keywords:** prolactin, polycystic ovarian syndrome (PCOS), hyperprolactinemia, insulin resistance, obesity, prediabetes, impaired fasting glucose

## Abstract

Polycystic ovarian syndrome (PCOS) is a prevalent endocrine condition in women of reproductive age, characterized also by insulin resistance, affecting both obese and non-obese individuals. Hyperprolactinemia in patients with PCOS may additionally aggravate the decline in insulin sensitivity, attributable to prolactin lipogenic effects and influence on metabolic profile. Therefore, this study aimed to investigate the serum levels of prolactin in women with PCOS and their associations with obesity, insulin resistance and prediabetes. A retrospective monocentric study was performed using the electronic database of 157 women diagnosed with PCOS. Serum prolactin, BMI, complete glucose-insulin profile and insulin resistance indices following OGTT were determined. The women with hyperprolactinemia (40.8%) had significantly higher BMI (*p* = 0.007), fasting glucose (*p* = 0.003), insulin levels (*p* < 0.001) and HOMA-IR (*p* < 0.001). The women with PCOS categorized as overweight/obese (47.1%), insulin resistant (68.8%), having impaired fasting glycaemia (28.7%) and prediabetes (36.3%) showed significantly higher levels of prolactin compared to the respective counterparts. Consequently, higher prolactin levels were significantly associated with an elevated risk of development of overweight/obesity (OR 2.59; 95% CI: 1.34–4.97, *p* = 0.004), insulin resistance (OR 3.33; 95% CI: 1.54–7.19, *p* = 0.002) and prediabetes (OR 1.98; 95% CI: 1.02–3.85, *p* = 0.043) in women with PCOS. Our results suggest that hyperprolactinemia might be a pathophysiological link between obesity, insulin resistance, and carbohydrate metabolism impairments in patients with PCOS. Increased prolactin levels may serve as an additional indicator of insulin resistance and even further exacerbate it in women with PCOS.

## 1. Introduction

Polycystic ovarian syndrome (PCOS) is the most common endocrine disorder among women of reproductive age, with a prevalence ranging between 5–20% according to the diagnostic criteria applied (5–20% or 10–15% according to the Rotterdam criteria and An-drogen Excess Society, respectively) [1,2]. The diagnosis of PCOS is established when two of the following three criteria are fulfilled: chronic anovulation, hyperandrogenism (clini-cal or laboratory), and the presence of polycystic ovaries. Alongside clinical symptoms, serum levels of 17-hydroxyprogesterone, dehydroepiandrosterone sulfate (DHEAS), free testosterone, and anti-Müllerian hormone (AMH) should be measured to assist in the diagnosis of PCOS. The diagnosis is typically one of exclusion, confirmed after rejecting hyperprolactinemia, thyroid dysfunction, and diseases of the pituitary and adrenal glands. Polycystic ovarian syndrome is characterized by moderate to severe insulin resistance. Insulin resistance affects approximately 27% of general female population, whereas this percentage raises up to 50–80% in women with PCOS. Insulin resistance is present in 70–95% of women with PCOS and obesity, while in 30–75% of those with PCOS and normal body weight. Elevated insulin levels are both an indicator and the principal cause of PCOS [1,2,3,4]. Hyperinsulinemia significantly contributes to the onset of PCOS and an increase in androgen hormone levels in women. Insulin resistance can impair ovulation and elevate testosterone production. Therefore, insulin resistance is considered a pivotal link between obesity and menstrual disorders. Hyperinsulinemia directly influences androgen metabolism, hence enhancing androgen synthesis in the context of insulin resistance [2,3]. The fact that insulin resistance is so common in lean PCOS patients, underscores that obesity is not the sole contributor to metabolic disturbances in women with PCOS [5]. Consequently, women with PCOS have an elevated risk of developing prediabetes, gestational diabetes mellitus, and type 2 diabetes. It is widely recognized that dysglycemia is influenced by both modifiable and non-modifiable risk factors. Polycystic ovarian syndrome is regarded as a non-modifiable risk factor. Insulin resistance is acknowledged as the root cause of several metabolic diseases. This has led to the classification of various phenotypic categories of PCOS (A, B, C, D), determined by metabolic impairments and body weight, facilitating the categorization of at-risk women [6]. More than 50% of the women with PCOS experience impaired glucose tolerance (IGT), impaired fasting glycemia (IFG), or type 2 diabetes even before reaching 40 years of age [3,4,5,6]. Polycystic ovary syndrome is associated with a four- to seven-fold increased risk of cardiovascular disease and with up to three-fold elevated risk of endometrial cancer [1,2,3].

Obesity markedly modifies the clinical and laboratory profile in women with PCOS. Obesity not only diminishes fertility and exacerbates insulin resistance, but also elevates the risk of overt diabetes mellitus. Insulin resistance is a significant, but not obligatory, characteristic in women with PCOS. Obesity is a thoroughly examined factor influencing the correlation between insulin resistance and PCOS. Conversely, numerous women with PCOS are not obese yet, but exhibit insulin resistance. Hence, it is crucial to examine supplementary hormonal and metabolic factors that may be regarded as potential contributors to insulin resistance in women with PCOS [7].

Besides its function in reproduction and lactation, prolactin (PRL) affects metabolic processes, cancer, and the immune system [8,9]. Prolactin is predominantly produced by the pituitary gland, but also in the endometrium, decidua, brain, breast, and adipose tissue [8]. Hyperprolactinemia is the most prevalent endocrine condition associated with the hypothalamic-pituitary axis. Physiological, clinical, pharmacological, and genetic variables can all affect lactotroph cell secretion, leading to hyperprolactinemia. Due to numerous factors that affect both PCOS and hyperprolactinemia, the prevalence of hyperprolactinemia in women with PCOS reported in the literature varies widely, ranging from less than 5% to more than 65% [10]. Recent studies have demonstrated that hyperprolactinemia is prevalent among women with PCOS, suggesting a pathophysiological association between the two conditions. A slight elevation in serum PRL levels can be attributed to PCOS and disregarded. However, elevated PRL levels suppress ovulation and can be the cause of polycystic ovaries. Multiple explanations have been proposed regarding the pathophysiological connection between hyperprolactinemia and PCOS. The predominantly accepted etiology is hypothalamic-pituitary dysfunction. Dopamine inhibits prolactin secretion, while estradiol and thyrotropin-releasing hormone (TRH) augment it. Reduced dopaminergic regulation is observed in patients with PCOS, leading to elevated levels of both prolactin and luteinizing hormone (LH) [10]. Alternative credible hypotheses encompass obesity-related elevation of TRH, the impact of increased unopposed estrogen, hyperandrogenemia, and insulin resistance. Nonetheless, none of these concepts could be considered united due to discrepancies in hormone levels, clinical manifestations, and body mass index (obese versus non-obese) among patients with PCOS.

Prolactin is believed to affect the development and functionality of adipose tissue and pancreatic beta cells. PRL is not only an adipogenic hormone influencing adipose tissue function, but it is also synthesized by adipose tissue and exerts both autocrine and paracrine actions [8,9]. Therefore, a correlation between elevated PRL levels and peripheral insulin resistance has been recently suggested. Adipocytes, pancreatic beta cells, type 2 dopamine receptors, and dopamine are important players of the integral regulation of insulin action. Increased PRL levels disrupt glucose and insulin metabolism and are associated with reduced insulin sensitivity in peripheral tissues in both obese and non-obese patients [9,11]. There are different scientific explanations for this effect of prolactin. According to one hypothesis, glucose and PRL synergistically induce insulin gene transcription [9,11]. Another possible cause of insulin resistance in hyperprolactinemia is the down-regulation of insulin receptors [11]. Prolactin is also hypothesized to influence insulin sensitivity and metabolic homeostasis in adipose tissue. Increased plasma PRL levels have been associated with aggravated tissue insulin sensitivity [12,13,14]. However, there are also discrepancies in the literature about the relationship between PRL and insulin resistance in PCOS. A recent study has documented lower levels of prolactin and a negative correlation between prolactin and insulin resistance in infertile women with PCOS [15].

Therefore, the aim of the current study was to investigate the serum levels of prolactin in women with PCOS and their associations with obesity, insulin resistance and prediabetes.

## 2. Results

Median (interquartile range) prolactin levels in the whole-study group were 545 (296–718) mIU/L. The patients were divided in two groups having normoprolactinemia (59.2%) and hyperprolactinemia (40.8%) according to the reference range for serum prolactin levels of the respective laboratory (125–635 mIU/L). No of the enrolled women had prolactin levels below the lower reference limit. Median (interquartile range) PRL levels in the two subgroups with normoprolactinemia and hyperprolactinemia were 327 (235–494) mIU/L and 753 (688–896) mIU/L, respectively.

The results from the comparative analysis of the baseline clinical characteristics of the women with PCOS between the groups with normoprolactinemia and hyperprolactinemia are summarized in Table 1. There were no significant differences between the groups with normoprolactinemia and hyperprolactinemia in regards of age and serum levels of any of the studied hormones [follicle-stimulating hormone (FSH), LH, thyroid-stimulating hormone (TSH), AMH, testosterone, DHEAS, androstenedione and 17-OH progesterone]. However, the women with hyperprolactinemia had statistically significantly higher body mass index (BMI), fasting glucose and immunoreactive insulin (IRI) levels. Consequently, hyperprolactinemia group also had higher homeostasis model assessment of insulin resistance (HOMA-IR) while only a tendency for increased homeostatic model assessment of β-cell function (HOMA-B). All these significant differences in BMI, fasting glucose, insulin levels and HOMA-IR were preserved (*p* < 0.05) even after the exclusion of the 9 (5.7%) women having adenoma from the hyperprolactinemia group. Although the median values of 1- and 2-h plasma glucose levels following the oral glucose tolerance tests (OGTT) were higher in the group with hyperprolactinemia, there was no statistical difference between the compared cohorts (Table 1).

In the whole-study group (n = 157), prolactin levels did not correlate statistically significantly with age or any of the hormones tested. There was only a slight tendency for a negative correlation between PRL and LH. However, a positive statistically significant correlation was registered between prolactin concentration and BMI (Table 2).

**Figure 1:**
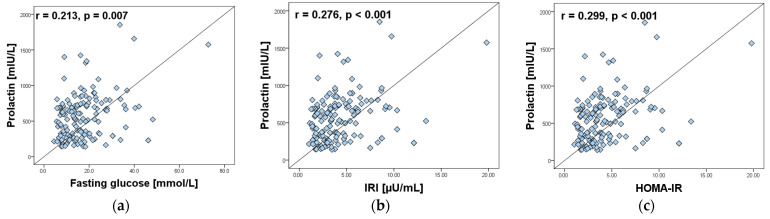
Correlations of serum prolactin levels in women with PCOS (n = 157) with: (**a**) fasting glucose; (**b**) IRI; (**c**) HOMA-IR.

In addition, PRL levels correlated positively and statistically significantly with fasting glucose, IRI and HOMA-IR index (Figure 1), but did not correlate with HOMA-B (r = 0.132, *p* = 0.101, n = 157).

Only LH correlated and tended to correlate negatively with fasting glucose (r = −0.165, *p* = 0.039) and 1-h plasma glucose levels post the OGTT (r = −0.153, *p* = 0.055) in the whole-study group (n = 157), respectively. No other statistically significant correlations were registered in the studied population with PCOS between the tested hormones and the parameters of glucose and insulin homeostasis.

Although, it was not a focus of our study, other correlations were also observed in the examined women with PCOS (n = 157). AMH correlated statistically significantly and positively with LH (r = 0.249, *p* = 0.002) and DHEAS (r = 0.174, *p* = 0.03). A positive correlation was registered between LH and FSH (r = 0.362, *p* = 0.008). As expected, testosterone correlated statistically significantly and positively with DHEAS (r = 0.373, *p* < 0.001), and androstenedione (r = 0.198, *p* = 0.013).

**Figure 2:**
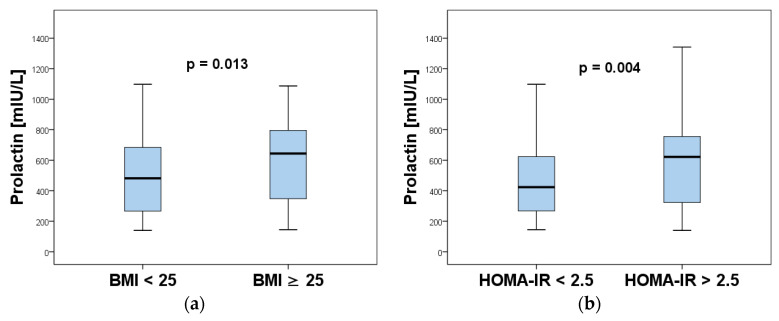
Comparison of serum prolactin levels in women with PCOS according to: (**a**) BMI (BMI < 25 and BMI ≥ 25; (**b**) insulin resistance (HOMA-IR < 2.5 and HOMA-IR ≥ 2.5).

Based on the significant correlations of PRL with BMI and HOMA-IR, we wanted to further investigate whether there was also a significant difference in prolactin levels in the women with PCOS with and without overweight/obesity and insulin resistance, respectively. Almost one half (n = 74, 47.1%) of the examined women with PCOS were categorized as overweight/obese (BMI ≥ 25). They had significantly higher PRL levels in comparison to their 83 (52.9%) counterparts with BMI < 25 (Figure 2a). According to the HOMA-IR index, 108 (68.8%) of the investigated women with PCOS had insulin resistance. Prolactin concentration was significantly higher in the subgroup with PCOS with insulin resistance (HOMA-IR > 2.5) than the subgroup with PCOS without insulin resistance (HOMA-IR < 2.5, n = 49) (Figure 2b).

**Figure 3:**
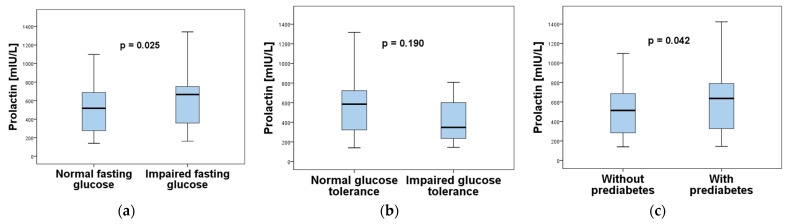
Comparison of serum prolactin levels in women with PCOS according to: (**a**) fasting glucose; (**b**) glucose tolerance; (**c**) presence of prediabetes.

According to the American Diabetes Association PCOS is as one of the risk factors requiring testing for prediabetes in overweight/obese women [16]. Therefore, we wanted to evaluate the prolactin levels according to the presence of IFG, IGT and prediabetes (defined as IFG and/or IGT). The obtained results are illustrated in Figure 3. Forty-five (28.7%) of the women with PCOS had impaired fasting glycaemia. They had significantly higher PRL levels than the women with fasting normoglycemia (n = 112, 71.3%) (Figure 3a). However, there was no significant difference in PRL levels between women with PCOS with impaired (n = 18, 11.5%) and normal glucose tolerance (n = 139, 88.5%) (Figure 3b). The criteria for prediabetes were met in 57 (36.3%) of the examined women with PCOS. They had significantly higher prolactin levels than their counterparts without prediabetes (n = 100, 63.7%) (Figure 3c).

A logistic regression analysis was further conducted to evaluate the associations between the increased prolactin levels and relative risk for the overweight/obesity, insulin resistance and prediabetes in the studied women with PCOS. Our results shown that hyperprolactinemia in women with PCOS was statistically significantly associated with 2.5 and 3.3 higher risk of development of obesity and insulin resistance, respectively. In addition, almost doubled was the relative risk of development of prediabetes in the women with PCOS and hyperprolactinemia (Table 3).

## 3. Discussion

Polycystic ovary syndrome is a complex condition with no distinctly identified etiological element associated with its manifestation. The clinical presentation is extremely various, often requiring reliance on laboratory markers for the diagnosis in many individuals. Multiple factors—genetic, environmental, hormonal, metabolic, etc.—may be considered in the overall etiology of the disease. Although, the hormonal alterations characteristic for the condition have been extensively documented, the relationship between hormonal and metabolic irregularities in PCOS is still inadequately comprehended. PCOS is classified as a metabolic disorder that elevates the risk of prediabetes, sleep apnea, type 2 diabetes, and cardiovascular disease [1,2,3]. The primary metabolic features of PCOS are insulin resistance and obesity, which are present in the majority of PCOS women. Nonetheless, diminished insulin sensitivity is also observed in lean individuals with PCOS [1,2,3,4,5]. Hyperinsulinemia significantly contributes to the progression from prediabetes to type 2 diabetes mellitus, as well as to the development of arterial hypertension and dyslipidemia, thus enhancing cardiovascular risk. Metabolic abnormalities impact more than 30% of women diagnosed with PCOS. Special attention is needed since women with PCOS have been shown to experience a faster transition from normal glycemia or dysglycemia to type 2 diabetes mellitus [4,17,18].

On the other hand, beyond its well-established effects on gonadal function, reproduction, and lactation, PRL has recently been recognized for its important role in metabolism. Prolactin promotes insulin secretion and is associated with both peripheral and hepatic insulin sensitivity [9,19,20]. Hyperinsulinemia and hyperprolactinemia frequently appear as laboratory test results in women diagnosed with PCOS. Hyperinsulinemia is a prevalent laboratory observation in women with PCOS, irrespective of their BMI. In women with polycystic ovaries, regardless of body weight, peripheral glucose clearance is diminished by approximately 40%, with obesity aggravating this condition [5]. Insulin resistance is consistently observed in patients with PCOS, as a significant majority of these individuals are obese. However, hyperinsulinemia is also prevalent in lean women with PCOS [5], and the underlying cause of its occurrence remains incompletely elucidated. Additionally, insulin resistance is known to exacerbate the PCOS complications.

Considering all the aforementioned, we aimed to investigate the serum levels of PRL in women with PCOS and their associations with obesity, insulin resistance and prediabetes. We registered normoprolactinemia in 59.2% and hyperprolactinemia in 40.8% of our study group comprised of Bulgarian women with PCOS. These results are in complete accordance with the ones obtained by Davoudi et al. They detected normal prolactin level in 63% and hyperprolactinemia in 37% of 330 Iranian women with PCOS. However, in contrast to our results, higher levels of LH were found in the women with normoprolactinemia [21]. A frequency of hyperprolactinemia only of 17.1% was found in newly diagnosed women with PCOS in Bangladesh [22]. Reports on the prevalence of hyperprolactinemia in PCOS vary widely in the literature, with estimates ranging from 3% to 67% [23]. These differences could be attributed not only to the different diagnostic criteria for PCOS, but also to some genetic factors. Saei Ghare Naz et al. showed that Eurasian PCOS patients had significantly higher prolactin levels, whereas PCOS patients from the African population had lower prolactin levels [24]. In addition, more accurate results from a study investigating the circadian prolactin profile, but not only single prolactin measurement in PCOS patients, showed no association between PCOS and elevated serum prolactin levels. Furthermore, the study revealed that the frequency of hyperprolactinemia occurrence is equal in women with and without PCOS. Therefore, Szosland et al. suggested that high prolactin is not a characteristic feature of PCOS. However, the importance of assessing serum prolactin levels in individuals with PCOS has been emphasized [25].

The primary objective of our study was to investigate the possible association between hyperprolactinemia, as part of the laboratory profile in PCOS, body mass index, and parameters of glucose homeostasis. The comparative analysis indicated that patients with hyperprolactinemia had significantly higher body mass index, fasting plasma glucose, immunoreactive insulin, and HOMA-IR than normoprolactinemic patients. Consequently, the correlation analysis demonstrated that serum prolactin levels correlated statistically significantly and positively with BMI, fasting glucose, insulin, and HOMA-IR. In contrast to our results, a retrospective analysis of 840 patients with PCOS in Bangladesh reported that PRL correlated negatively with age, BMI, waist circumference and the presence of metabolic syndrome [22]. Another study has also shown that prolactin correlated negatively with HOMA-IR and HOMA-B in infertile women with PCOS [15]. The relationship between prolactin levels and BMI in samples from general population is also rather controversial and most of the studies have documented lack of correlation in healthy subjects [26]. However, according to Al Sabieh et al. weight gain may be regarded as an independent symptom in individuals with hyperprolactinemia, and that these patients exhibit a higher prevalence of obesity [27]. These discrepancies could be due to the fact that both high and very low PRL levels have been recently linked to negative metabolic outcomes, while moderately elevated PRL levels have beneficial effects on various metabolic aspects, including insulin resistance [15,28]. Insulin resistance is consistently observed in patients with PCOS, as a significant majority of these individuals are obese, but insulin resistance is also prevalent in lean women with PCOS. In coincidence with this, in our study almost 50% of the examined women with PCOS were categorized as overweight/obese, while greater percentage (69%) of the women with PCOS had insulin resistance. Consequently, overweight/obese patients with PCOS showed elevated PRL levels in comparison to individuals with normal body weight. Similarly, the women with PCOS who had insulin resistance also exhibited higher prolactin levels than their counterparts without insulin resistance. Therefore, high prolactin levels may be regarded as an additional factor worsening the glucose-insulin profile in PCOS.

These findings could be explained with the additional physiological effects of prolactin. Elevated prolactin levels can modify central appetite centers, increasing hunger sensations, augmenting food consumption, and thus resulting in weight gain. Furthermore, prolactin is a lipogenic hormone, and elevated levels can result in obesity, subsequently impacting peripheral insulin sensitivity [7]. Prolactin can serve as a regulator of insulin sensitivity in peripheral and fat tissues. The results of studies conducted on women with hyperprolactinemia further corroborate this. They exhibit reduced insulin sensitivity in comparison to women with normal hormone levels [29,30]. Delcour et al. also showed that abnormally high serum PRL levels contribute to metabolic comorbidities and infertility in women with PCOS [23]. Other studies have also shown a relationship between elevated serum PRL levels and the resistance of peripheral tissues to insulin [12]. According to Goyal et al., both PCOS and hyperprolactinemia induced hyperinsulinemia [31]. Increased PRL levels could induce alterations in the glucose profile, resulting in diminished glucose tolerance and insulin sensitivity [9,20].

Consequently, in obese women with polycystic ovaries, prolactin and insulin may serve as potential diagnostic indicators of PCOS. Therefore, hyperprolactinemia might be an additional link between obesity and insulin resistance. Indeed, our further logistic regression analysis showed that hyperprolactinemia was associated with a 2.5 and 3.3-times increased risk of obesity and insulin resistance, respectively.

Hyperprolactinemia and hyperinsulinemia, whether pre-existing or generated by elevated prolactin levels, may be regarded as risk factors for the development of carbohydrate metabolism problems. The association between hyperprolactinemia and poor glucose tolerance, as well as type 2 diabetes mellitus, is evidenced by data from a population study conducted by Wang T et al. [32]. Given the augmented risk of dysglycemia in women with PCOS, along with its influence on body weight and insulin resistance, we conducted a further analysis of the association between hyperprolactinemia, and the incidence of prediabetes in women with PCOS. Our results indicated that the relative risk of prediabetes is nearly doubled in women with PCOS and hyperprolactinemia. To the best of our knowledge this is the first study estimating the association between prolactin levels and prediabetes in PCOS. The involvement of PRL as pathophysiological intricate link between prediabetes and PCOS has been recently confirmed by Krysiak et al. [33]. Their study investigated the effect of metformin and included patients with both hyperprolactinemia and prediabetes, with and without PCOS. While metformin improved insulin sensitivity in both groups, the effect was more pronounced in those without PCOS. Notably, a reduction in prolactin levels was observed only in PCOS patients [33]. All this has confirmed that hyperprolactinemia might worsen and speed up the manifestation of PCOS metabolic complications and could make them even more difficult for treatment. Administering dopamine agonists to patients with hyperprolactinemia is an effective therapeutic strategy, as it not only lowers excessive prolactin levels but also resolves metabolic disorders [34]. Dopamine agonist therapy has been demonstrated to influence carbohydrate metabolism and body weight, but this class of drugs is not considered first-line treatment for obesity and carbohydrate metabolism impairments [35]. A meta-analysis conducted by Byberg et al. showed a decrease in weight, along with an improved lipid profile and glucose tolerance, following therapy with dopamine agonists for patients with prolactinomas [36].

It is intriguing to ascertain whether a restoration of prolactin levels will beneficially influence not only ovulatory disorders but also metabolic conditions. A prospective evaluation of patients with hyperprolactinemia, obesity, and carbohydrate impairments undergoing treatment with dopamine agonists is warranted.

The study has several limitations. The study is retrospective; however, in a prospective series, the outcomes following the implementation of therapy might be assessed. Furthermore, causality cannot be drawn from simple correlations in a retrospective study. Macroprolactin levels were not assessed. Not all patients received nuclear magnetic resonance imaging of the pituitary gland to rule out a tumor associated with hyperprolactinemia.

## 4. Materials and Methods

### 4.1. Study Design and Setting

A retrospective monocentric analysis utilized the electronic database of the Specialized Hospital for Obstetrics and Gynecology “Dr. Shterev”, Sofia, Bulgaria. Ethical approval was received from the Institutional Review Board of the hospital.

### 4.2. Study Population

This retrospective cohort study included 157 women with PCOS with mean age 28.0 ± 5.2 years and BMI = 26.9 ± 6.8 kg/m^2^. PCOS was diagnosed using the Rotterdam criteria for diagnosis, which were endorsed by the Androgen Society. Baseline levels of FSH, LH, PRL, estradiol, testosterone, DHEAS, 17-OH-progesterone, and AMH were measured in all patients between the third and the fifth days of spontaneous, non-drug-induced menstruation. The study included data from patients who had not used hormonal treatments, insulin sensitizers, dopamine agonists, other insulin action mediators or any other drugs at the time of the study. Two groups of individuals were identified: those with hyperprolactinemia (40.8%) and those with normoprolactinemia (59.2%). Patients in both groups were classified as obese, overweight, and normal-weight based on BMI values of ≥30, 25–30, and <25, respectively [37]. Only 9 (5.7%) of the patients were diagnosed with adenoma.

All patients performed one-step 75-g OGTT, with measurement of the blood glucose levels on 0, 60 and 120 min. Along with glucose, the IRI were evaluated and HOMA-IR index was calculated. The index of homeostasis model assessment of insulin resistance (HOMA-IR) was calculated as fasting serum insulin (μIU/mL) × Fasting glucose (mmol/L)/22.5. The index of HOMA of β-cell function (HOMA-B) was calculated as (20 × fasting serum insulin)/(Fasting glucose − 3.5). Impaired fasting glucose was defined as fasting plasma glucose values of 5.6 to 6.9 mmol/L. Impaired glucose tolerance was defined as two-hour plasma glucose levels of 7.8 to 11.0 mmol/L on the OGTT. Prediabetes was defined by the presence of IFG and/or IGT [16].

### 4.3. Laboratory Analysis

Blood samples were collected for hormone analysis during the early follicular phase (between the third and fifth day of the period), after at least 12 h of overnight fasting between 8:00 and 9:00 a.m. Prolactin, FSH, LH, estradiol, testosterone, 17-OH-progesteron, DHEAS, androstendion, AMH and TSH levels were determined using the Immunochemical analysis ECLIA (Roche Cobas 8000, Basel, Switzerland), with the laboratory’s reference interval being: PRL—125–625 mIU/L; FSH—3.5–12.5 mIU/L (early follicular phase); LH—2.4–12.6 mIU/L (early follicular phase), Estradiol—45.4–854 pmol/L (early follicular phase); testosterone—0.22–2.9 nmol/L; androstendion—4.29–13.68 nmol/L; DHEAS—13.3 nmol/L; 17-OH-progesteron—0.3–2.4 nmol/L (early follicular phase); AMH—1–6.8 ng/mL; TSH—0.3–4.20 mIU/L. Immunoreactive insulin (IRI) levels were assessed using the electrochemiluminescence method (Roche Cobas 8000, Switzerland) with reference intervals for the respective laboratory of 2.6–24.9 mIU/L. The UV hexokinase method (IFCC) was used to assess plasma glucose levels during OGTT, with a reference range of 3.6–6.1 mmol/L. HOMA-IR < 2.5 was adopted as normal and HOMA-IR ≥ 2.5 was classified as insulin resistance [16].

### 4.4. Statistic Analysis

Statistical analysis was performed using SPSS software, version 20.0 (SPSS Inc., Chicago, IL, USA). Normal distribution was evaluated using the Kolmogorov-Smirnov test. Continuous variables with normal distribution were expressed as mean ± SD. Skewed distributed data were expressed as median and interquartile range (25th-percentile–75th-percentile). Student’s *t*-test or Mann-Whitney U test was used to compare two groups with Gaussian or non-Gaussian distribution, respectively. Spearman’s correlation test was performed to evaluate the correlations between the studied parameters. A binary logistic regression analysis was conducted to assess the associations between prolactin and the risk of obesity, insulin resistance and prediabetes. The results were expressed as odds ratios (OR) with 95% confidence intervals (95% CI). Differences were regarded as statistically significant at *p*-value < 0.05.

## 5. Conclusions

The results of the present study suggest that hyperprolactinemia may be a pathophysiological link between obesity, insulin resistance, and carbohydrate metabolism impairments in patients with PCOS. Increased prolactin levels may serve as a further marker of insulin resistance in women with polycystic ovary syndrome. Hyperprolactinemia and hyperinsulinemia might synergistically influence carbohydrate metabolism, elevating the risk of fasting dysglycemia and prediabetes. Further studies are warranted to confirm these associations.

## Figures and Tables

**Table 1:** Demographic, clinical and laboratory characteristics of the women with PCOS subdivided according to the serum prolactin levels.

Characteristics	Normoprolactinemia (*n* = 93)	Hyperprolactinemia *(n* = 64)	*p*-Value
Age (years)	28.9 ± 5.0	28.7 ± 5.4	0.699
Body mass index (kg/m^2^)	24.0 (21.0–30.0)	28.0 (23.5–33.8)	0.007 *
FSH (mIU/L)	5.78 (4.90–6.68)	5.32 (4.6–6.49)	0.222
LH (mIU/L)	9.90 (8.42–13.40)	9.38 (7.19–11.01)	0.104
TSH (mIU/L)	2.50 (1.42–3.41)	2.39 (1.69–3.40)	0.933
AMH (ng/mL)	7.90 (6.12–10.30)	7.23(6.18–9.01)	0.358
Estradiol (pmol/L)	142.0 (93.0–171.0)	137.0 (97.3–204.5	0.571
Testosterone (nmol/L)	1.60 (1.12–2.10)	1.57 (1.18–1.98)	0.513
DHEAS (nmol/L)	8.81 (6.87–11.20)	9.50 (6.74–12.30)	0.342
Androstenedione (nmol/L)	5.87 (3.65–8.56)	6.17 (4.21–8.16)	0.751
17-OH Progesterone (nmol/L)	2.12 (1.25–3.24)	2.33 (1.64–3.70)	0.138
Fasting glucose (mmol/L)	5.23 ± 0.55	5.48 ± 0.47	0.003 *
1-h plasma glucose post-OGTT (mmol/L)	7.25 (5.90–8.78)	7.44 (5.47–8.65)	0.686
2-h plasma glucose post-OGTT (mmol/L)	5.98 (5.12–6.84)	6.11 (5.10–7.01)	0.939
IRI (mIU/L)	13.40 (8.51–17.98)	18.48 (13.25–26.35)	<0.001 *
HOMA-IR	2.97 (1.84–4.11)	4.40 (3.02–6.29)	<0.001 *
HOMA-B	154.6 (104.5–232.3)	172.4 (133.9–263.3)	0.083

Data are presented as mean ± SD or median (25th—75th percentile). * indicates statistical significance.

**Table 2:** Correlations of serum PRL with age, BMI and hormonal parameters in women with PCOS (n = 157).

	Prolactin	
Characteristics	Correlation Coefficient (r)	*p*-Value
Age	−0.033	0.680
BMI	0.180	0.024 *
FSH	−0.087	0.280
LH	−0.140	0.081
TSH	−0.003	0.966
AMH	−0.004	0.964
Estradiol	0.050	0.536
Testosterone	0.006	0.938
DHEAS	0.053	0.510
Androstenedione	−0.033	0.685
17-OH Progesterone	0.067	0.402

*—statistical significance.

**Table 3:** Unadjusted associations between hyperprolactinemia and relative risk for overweight/obesity, insulin resistance and prediabetes in women with PCOS (n = 157).

	Hyperprolactinemia		
Characteristics	Unadjusted Odds Ratio (95% CI)	Chi-Square	*p*-Value
Overweight/Obesity	2.59 (1.34–4.97)	8.26	0.004
Insulin resistance	3.33 (1.54–7.19)	9.90	0.002
Prediabetes	1.98 (1.02–3.85)	4.11	0.043

## Data Availability

The data presented in this study are available upon reasonable request from the corresponding author.
